# Pun processing in advertising posters: evidence from eye tracking

**DOI:** 10.16910/jemr.16.3.5

**Published:** 2023-12-31

**Authors:** Anastasiia Konovalova, Tatiana Petrova

**Affiliations:** St. Petersburg State University, Russia

**Keywords:** Eye movement, eye tracking, attention, intermodal processing, pun, polycode text, advertising posters, ambiguity

## Abstract

This study examines the process of reading polycode advertising posters, focusing in particular on the effect of a pun in the headline. The pun, or a sequence of lexical items that can be perceived as ambiguous, is contained in the headline and different meanings of this sequence are supported by the picture and text. The results of the preliminary experiment showed that advertisements with puns are rated as more attractive, original, effective and positive compared to advertisements without puns. We hypothesized that puns in the headlines increase cognitive effort in processing posters, leading to higher evaluations. The main experiment tested this and examined differences in eye movement when reading posters with and without puns. Fifty-five Russian participants viewed advertisements while their eye movements were recorded. Our results showed no fundamental differences in the general pattern of viewing advertisement posters with and without puns. We found that readers start to perceive polycode advertisements from the text and spend more time reading the text than looking at an image. These findings shed light on how attention is distributed between verbal and non-verbal components of polycode texts, and which type of poster is more effective for information retrieval at different processing levels.

## Introduction

Ambiguity is one of the properties of human language as a natural sign system. We encounter ambiguous and indefinite information all the time. A person perceiving speech or text constantly makes predictions about the content of what they have heard or read based on the existing context and their previous language experience. Ambiguity is present at all language levels and has been the subject of linguistic studies for many years. These studies examine why ambiguity appears in speech, how ambiguous words or phrases are used, and what factors influence the choice of a particular meaning from among several (see the review of 11). Ambiguity may arise when comparing the verbal and nonverbal parts of a polycode text – a text that includes picture and text components. This article investigates viewing processing of verbal and non-verbal information to resolve ambiguity in advertising posters within the eye-tracking paradigm.

## Background

### Image–text relations in a polycode text

Multimodal and polycode texts integrating both verbal and non-verbal types of information have become an important part of our everyday life. Mayer's (35) Cognitive Theory of Multimedia Learning suggests that effective engagement with the material presented in the form of polymodal text occurs due to the need to switch attention between text and image, oral signal and text, and establish the connection between these elements. Within the scope of this study, we need to distinguish the definitions of multimodal and polycode texts. Multimodal texts can include written text, visual imagery, audio, spatial arrangements, and gestures. Polycode text is a combination of verbal and non-verbal visual parts, it involves “a complex interplay of written text, images and other graphic elements” (28: p. 15). Text and image in a polycode text can be in equal relations, being independent or complementing each other, or they can be in unequal relations (classification by 33). Within unequal relations, two types of intersemiotic relationships are considered, namely image-subordinate-to-text and text-subordinate-to-image. When image is subordinate to text, image is related to only a part of text, and when text is subordinate to image, text may well be related to only a part of image. Thus, image and text go together to make meanings, and their differences can be utilized.

A special type of relationship between text and image in a polycode text is ambiguity or a conflict between its verbal and non-verbal parts. One of the most typical examples of such a text is a polycode advertising poster. Most often it consists of a text and an image (picture) and is found everywhere: in public transport, on billboards, on the Internet, etc. A huge number of linguistic means are used in advertising text, one of which, quite popular, is the use of intentional ambiguity or punning wordplay (52; 30; 14). Intentional, or deliberate, ambiguity refers to the ability of a single phrase to be interpreted in more than one way. Intentional ambiguity in headlines has a positive effect on ad evaluation (30), but it is still unknown which cognitive mechanisms ensure higher evaluation of ads containing ambiguity or puns.

### A pun as a way to construct a polycode advertising poster

A pun is a joke based on the semantic unification in one context of either different meanings of one word, or different words (phrases) that sound identical or similar (46). However, a word (phrase) with several meanings does not automatically make a pun (38: p. 1795). For ambiguity to turn into a wordplay, two conditions should be met: 1) meanings are opposing each other; 2) ambiguity is intentional. Thus, all puns are intentional and are based on several meanings of the same word (phrase) colliding with each other. Both polysemous words and collocations are often used for this purpose. Collocations are “lexically and/or pragmatically constrained recurrent co-occurences of at least two lexical items which are in a direct syntactic relation with each other” (5: p. 76).

There are two principles of language organization: idiom, or collocational, and open-choice (50). According to the idiom principle, by default we interpret the discourse as consisting of collocations. However, if this principle fails us (= we do not understand the meaning of the discourse), we proceed to interpret the discourse item by item. In case of collocations, each collocation is relexicalized, and we reinterpret it literally. Puns are rooted in such a forced switch from the idiom principle of interpretation to the open-choice principle.

L. Lagerwerf (30) gives an example of a pun used in an election campaign advertisement of Ken Livingstone (England). The poster shows Ken among several other people standing in a subway train carriage. The slogan reads, “Where Ken stands on the subway”. The interpretation of the slogan is deliberately ambiguous. The humor lies in the reflexive use of the phrase to stand on something, which means “to have a (strong) opinion”. The picture of Ken suggests that this phrase can be understood not as a collocation, but as separate lexical units stand + on + the tube. As illustrated by this example, a pun can be created through both verbal and nonverbal (pictorial) forms of communication.

The creator of a pun expects that upon hearing a sequence of sounds (or reading a sequence of letters), the addressee understands this sequence in the meaning (1), and the semantic field of the meaning (1) is activated for them. Only after that, a context appears in which the same sequence is understood in a different meaning (2) (38). Resolving a pun requires from the reader a cognitive effort (52). Processing of idiomatic expressions can be influenced by their frequency, “familiarity”, perceptual salience of one of the meanings, decomposability (i.e., motivation transparency for a native speaker), presence of a favorable context, and other factors (see 51). The author of the pun needs to be sure that the addressee will understand the pun, i.e. take into account all these factors beforehand.

There is a trend to include different forms of wordplay (for instance, a pun in a headline) into advertising posters as well as to study how it influences the accuracy of understanding and recognition. The current marketing demand is “saying more with less” (42), and the use of puns in the text of advertidements serves it well. Ambiguity resolution in reading polycode texts is particularly interesting as a research topic.

In a preliminary experiment (see (27) for details), we tested whether a pun in the headline of a polycode advertisement makes it more attractive and interesting for the reader/viewer. The results showed that advertising posters with puns were rated as significantly more attractive, original, effective and evoking positive emotions than posters without puns. Moreover, ambiguous posters were rated as more comprehensible than unambiguous posters, despite the fact that wordplay was expected to make ambiguous posters more difficult to understand. In the recognition task, we found that both verbal and nonverbal components of a poster are recognized better if they are part of an ambiguous poster.

The purpose of this study is to verify preliminary results and provide eye-tracking data on the processing of advertising posters.

### Eye-tracking as a method to study the processing of polycode texts

The eye-tracking method has been widely used to describe the visual processing of linguistic information at different levels: graphical, lexical, syntactic (31), at the level of perception of ambiguous phrases (24; 51) and to investigate the processing of polycode and multimodal texts of different genres. These genres include educational multimodal texts (see the reviews of 47; 3), advertisements (see the reviews of 55; 18), graphs ( 2; 1), sketches, or visual notes (40), newspapers (22; 57), comics (26), websites (53; 19; 7), and more. Eye-tracking is employed to investigate the postulates proposed within the system-functional approach to multimodality (10), as well as to experimentally study the effect of image-text relation on multimodal texts perception (34).

Eye-tracking is widely used to identify how attention is distributed between verbal and nonverbal components of polycode texts (see 32; 39; 58). By measuring saccades, fixations and regressions, eye-tracking research can reveal problematic zones for a reader/viewer, and also answer the question when and why the difficulties occur and how a reader resolves them. It was shown that picture and written text presented together can contribute to better understanding of the information than if presented separately (48). According to Obermiller & Sawyer (37), processing of a polycode text starts with non-verbal parts. Rayner et al. (45) suggested that people spend more time processing the text than the picture.

The following questions are raised in the field of studying advertising perception: how readers integrate text and images in print media and how visual attention is distributed between advertising elements (21). Advertising perception is influenced by many factors (18), including: visual characteristics of the image, font and text size, instructions given to the participants of the experiment, etc. In advertising processing, the reader's attention is directed by top-down and bottom-up effects (55). In bottom-up processing, attention is drawn to prominent objects of the advertisement such as large text, bright colorful objects, etc. The reader's attention is involuntarily moved by these objects. On the other hand, top-down processing involves following the content of what is seen, driven by internal factors such as goals and expectations of a reader. This type of reading requires more effort and is slower. These viewing strategies work together.

Besides bottom-up and top-down factors, the viewing pattern is also influenced by central gaze bias (our eyes tend to gaze at and return to the center of the screen) and information maximization approach (our eyes may be spatially positioned to optimize the acquisition of visual information) (12). Furthermore, when perceiving a scene, human faces and text attract our gaze more than other visual objects (9). Human faces are considered to be “special stimuli” that tend to capture the visual attention of viewers (56: p. 6). Marketing research has shown that the presence of a human in a visual stimulus can influence viewing behavior (18).

In addition to visual characteristics, the perception of an advertising poster is influenced by the structural-semantic relationship between verbal and non-verbal components of the advertisements. Radach et al. (44) investigate the influence of the complexity of pragmatic image-text relations on advertising processing. Implicit advertisements that include complex relationships between image and text elements are viewed longer and rated higher than explicit advertisements that include a direct text-image relationship. Puškarević et al. (42) describe how visual complexity of advertisement affects its perception by viewers. They used advertisements in which slogans were visually amplified with a rhetorical figure using typeface design. The results showed that viewers pay more attention to the advertisement as a whole, but not to the brand name, when the rhetorical imagery of the typeface is used.

Eye-tracking studies on the processing of verbal and non-verbal elements in various polycode texts are currently relevant. To the best of our knowledge, this is the first time Russian-language material has been used for such a study.

This study examines advertising posters in which a pun is created using all the components of a polycode text, both verbal and pictorial. The pun itself, or a sequence of lexical items that can be perceived as ambiguous, is contained in the headline of an advertising poster, and different meanings of this sequence are supported by the picture and text. Thus, the pun becomes polycode. This article presents the results of the eye-tracking experiment alongside a summary of a preliminary experiment, described in detail in (27). The description of the preliminary experiment helps to understand the goals of the eye-tracking experiment. Results from the preliminary study show how the readers/viewers comprehend and evaluate advertising posters at a high-level of processing. Eye-tracking provides an opportunity to examine low-level reading mechanisms when viewing posters. To reveal if there are differences between lower perceptual processes and high-level comprehension processes, we conducted an eye-tracking experiment.

### Research questions

The main goal of this study is to investigate through eye-tracking the processing of polycode posters containing a pun in an advertising headline leading to a conflict between verbal and nonverbal components of the poster. The study aims to address three research questions: (1) What is the impact of puns in advertising posters on eye movement patterns?; (2) How does the viewing of verbal and non-verbal components of advertising poster differ?; and (3) Are there any differences between lower perceptual processes (parameters of eye movements) and higher comprehension processes (subjective evaluation and recognition of advertising posters)?

## Material and Methods

### Hypothesis

Our hypothesis is that posters with puns and poster without puns are processed differently, namely, since the puns are polycode, readers will switch their gaze more often between text and picture on posters with puns than on posters without puns, reflecting the process of verbal and non-verbal information integration. The primary focus is to reveal if a reader/viewer processes posters without puns with less cognitive load than posters with puns. We hypothesized that the presence of a pun in the heading makes the information processing more resource-intensive and therefore leads to the better poster evaluation.

### Participants

55 native speakers of Russian participated in the experiment. Data from two participants (female and male) were excluded from the analysis due to the poor quality of the eye-movement data. Since the puns in the posters are often witty, participants smiled and squinted their eyes during the experiment, which resulted in loss of gaze on the eye-tracker. The rest of the participants successfully completed the tasks, so their data were used in the analysis. As a result, the final study sample consists of 53 participants (42 females, 13 males, aged from 18 to 35 years, Mage = 22, SD = 5,1). All participants had normal or corrected to normal vision. Each participant had provided written informed consent prior to participating in the experiment.

### Material

**Figure 1.**
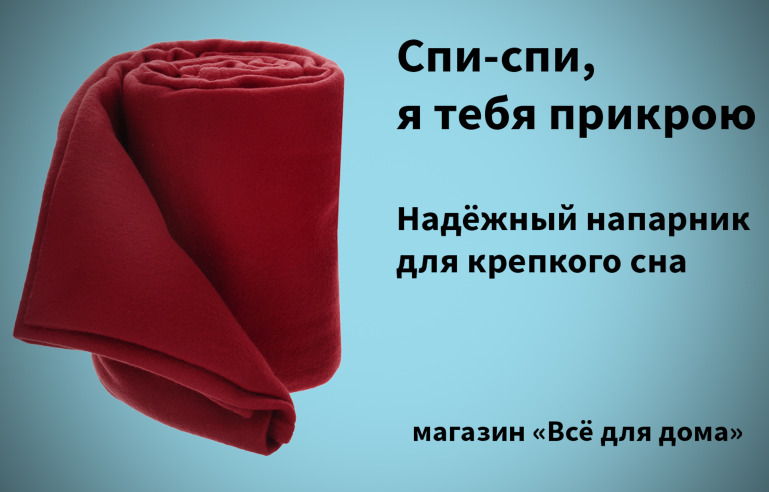
An advertising poster with a pun. The text says: Sleep, sleep, I'll cover you (headline). A reliable partner for good dreams (main advertising text). “Everything for housekeeping” shop (brand name). The collocation to cover (for) someone in the headline has two meanings: idiomatic ‘cover for someone’ and literal ‘cover something with something’.

We used 11 advertising posters with complex text-image relations. Examined posters contain a conflict between verbal and pictorial components of a polycode text. For example, in a poster advertising a blanket (see Fig. 1), the headline contains the collocation to cover (for) someone, which, when first read, is understood to mean “protect by military action (special)” (13), and the main advertising text supports this meaning with the word partner. However, the picture of a throw blanket supports the literal meaning of the collocation: “to cover (conv.)” (ibid.).

**Figure 2.**
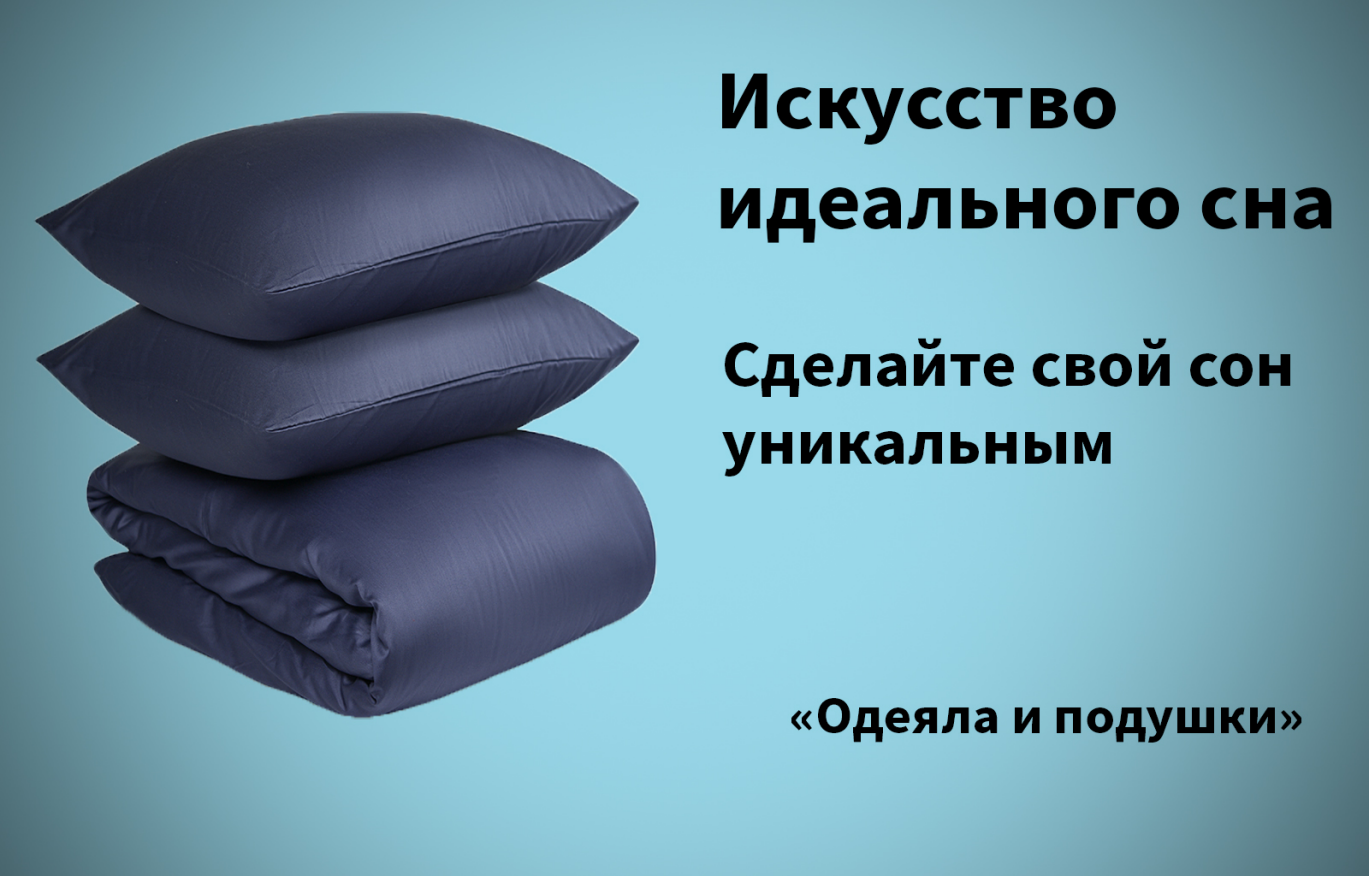
An advertising poster without a pun. The text says: The art of perfect sleep. Make your sleep unique. “Blankets and pillows”.

The study also featured 14 posters without puns. Most advertising posters were found online, although two posters without pun were created from scratch to pair with the pun posters. For an example of a poster without a pun, see the poster that is paired with the poster advertising the blanket (Fig. 2). All posters were edited to look uniform, i.e. aligned according to a number of parameters, which, as shown by numerous studies (see, for example, 18; 49), affect the perception of advertisements. Specifically, the posters were resized to 1600x1024 pixels, with the image occupying the left half of the poster. The verbal component of the posters consists of three text zones located in the right half of the poster. The headline occupies one or two lines within the range of 0 to 342 pixels, the main advertising text occupies two or three lines within the range of 342 to 684 pixels, and the brand name occupies one line within the range of 684 to 1024 pixels. The brand name is a conditional identifier of the third text zone. It either consists of a fictional abstract name for the advertised product (e.g., “Favorite” milk, “Fitness Club”) or simply states the fictional name of the store where the product can be purchased (e.g., “Tekhnika” store, “Everything for the home” store) in the case of commercial advertisements. In social advertisements, the brand name may indicate the advertiser, such as the Ministry of Health, or clearly state that it is a social advertisement. In the case of political advertising, the brand name indicates that it is related to municipal election.

Source Sans Pro Bold font was used for the text on the posters, and the font sizes were proportional for the three text zones (the largest for the title, the middle for the main advertising text and the smallest for the brand name). It was not possible to maintain the same font size for all text zones on all the posters, which is due to the attempt to position the text in these zones in the most convenient way. However, despite this variation, visually all the posters look the same, as the relative proportions between the font sizes of different text blocks are preserved.

The uneven number of posters with and without puns needs to be explained. There were the same number of posters of two types, namely 14 with puns and 14 without puns, when the study started (27). Initially, eye movement data was collected for all these 28 posters. Each poster with a pun had a paired poster without a pun promoting the same product or issue. The posters included 20 paired posters with commercial advertisements for fitness clubs, tablet plans, furniture, blankets, milk, cheese, laptops, car dealerships, and hardware stores. In addition, another six posters were social advertisements related to child care, bad habits and human health, and two posters were political advertisements dedicated to municipal elections. All posters had the same structure and the same picture-text layout, as previous studies (49) have shown that presentation format has a significant effect on eye movement metrics when reading on-screen text.

After all participants were recorded, a more detailed linguistic analysis of the stimulus material was conducted. This analysis revealed that not all puns in the posters were constructed in the same way. Some titles containing puns were based on playing with polysemous words rather than on the literal and idiomatic meanings of collocations. For example, one of the headlines read: “Which card is more important?” This headline played with two meanings of the word card: a credit card and a photocard. As a result, three posters with puns that relied on polysemous words rather than collocations were excluded from the analysis. These excluded posters advertised child care, human health, and a fitness club. However, it was decided to keep the three posters without the puns that were originally paired with the excluded posters.

We found pun-based posters on the Internet and did not modify the headlines. Therefore, we could not control factors such as collocation composition, length, and the frequency of words used in it. These unconsidered factors might affect the processing of the verbal part of the posters.

Because we did not edit the text or image on all but two of the posters, which we created from scratch, the non-verbal part of the posters is very diverse. The non-verbal component of 22 posters presents colorful, realistic depictions of people, objects, and animals. In two posters (hardware store advertisements), the non-verbal component is represented by a black abstract symbol (gender symbols and a schematic representation of a construction brush), and one poster features a colorful fictional creature: the udder of a running cow, represented as a four-legged animal that carries milk in it like a mug.

The non-verbal components of the advertising posters depict people (13 posters: 5 with and 8 without a pun), objects (8 posters: 3 with and 5 without a pun), abstract symbols (2 posters: 1 with and 1 without a pun), animals (1 poster with a pun) and a fictional creature (1 poster with a pun).

We found no statistically significant differences between the number of characters including punctuation marks and excluding spaces in each of the three text zones separately, nor in the total number of characters in the text for posters with and without a pun (see Table 1).

All materials used in the study can be found at the link to the OSF project: https://osf.io/te65g/.

**Table 1.** Mean values of the number of characters in the headline, main advertising text, and brand name of advertising posters, and the total number of characters in the text, as well as the p-value according to the Mann-Whitney criterion

	Posters with a pun	Posters without a pun	*p-value*
total N of characters (M)	57	59	0.25
N of characters in headlines (M)	16	19	0.108
N of characters in main advertising texts (M)	23	25	0.303
N of characters in brand names (M)	18	16	0.447

### Procedure

Participants' eye movements were recorded using the SR Eyelink1000 plus eye tracker (SR Research Ltd., ON, Canada). Stimuli were presented on a ViewSonic G90fB Graphics Series 19” CRT monitor (refresh rate 120 Hz, resolution 1600x1024 pix). Calibration consisting of 9 points was performed before the start of the experiment, drift correction was performed before the presentation of each subsequent poster. After successful calibration and validation, participants viewed the posters on a screen located at a distance of approximately 70 cm. Monocular mode and a headrest were used to record eye movements. We used the SR Research Experiment Builder to create and run the experiment.

The participants were asked to examine the posters and, after viewing each one, to rate how original and eye-catching the posters were, and to answer whether the statement they saw on the screen matched the content of the poster. The following instructions were given to the participants:

You will see advertising posters. Your task is to carefully study each poster that appears on the screen and rate it on a scale of 1 to 5 by verbally answering the following questions:

1) Does this poster attract attention? (Would you pay attention to it?)

2) Rate this poster on a level of originality? (Consider the headline, text components, composition, etc.)

After evaluating the poster, press any button on the joystick, and a statement will appear on the screen. Determine whether or not the statement corresponds to the content of the poster.

If this statement matches the content of the poster, press the Green button.

If this statement does not match the content of the poster, press the Red button.

This task is not timed. There are 28 posters in total.

There were 14 statements related to the content of the verbal part of the posters (e.g., “The text of the advertisement suggests undergoing a medical examination,” “The headline implies sitting comfortably in an armchair, covered with a blanket”). Half of these statements corresponded to the content of the advertising text, and the other half did not. The remaining 14 statements referred to the non-verbal part of the poster (e.g., “The woman on the poster is doing squats”, “The poster shows a football goalkeeper trying to catch a laptop”). Again, half of these statements matched to the content of the non-verbal part, and the other half did not. This task was given to the participants to get them more engaged with the posters and to keep them interested while viewing the stimuli.

To ensure that participants were on an equal footing before viewing the posters, before viewing the first and each subsequent poster, they were asked to press any button on the joystick, after which a black dot appeared on the screen. Participants were instructed to focus on the dot. The experimenter then started the stimulus display.

Before proceeding to the experiment, the participants trained on two advertising posters that were excluded from the analysis. We did not limit the time participants spent viewing the posters. All the stimuli were presented in random order. On average, the experiment took 15–20 minutes.

## Results

Four areas of interest (AOI) were defined for each poster: picture, headline, main advertising text, and brand name. The following eye movement measures were analyzed: total dwell time (the sum of all fixation durations in the area), first run dwell time (the sum of fixation durations in the area before the gaze moves to another area), fixation count, regression count, run count between areas. Total dwell time is traditionally used for studying multimodal text processing (40) and is considered to be a marker of attention to AOI content (23). First-run dwell time may indicate early processing and object recognition; this measure increases for semantically informative objects (20). Fixation count on AOI has been used as an indication of semantic importance (20) and may indicate the intensity of processing (3). Regression count may indicate difficulties that the reader faces when trying to understand a particular fragment of the material (16). Run count between AOI indicates that the integration process is happening (3), which can manifest itself in an increase in the number of transitions between AOIs such as text and pictures (21; 3).

The first fixation, associated with the fact that before viewing the stimuli, participants looked at a point in the center of the screen, most often not falling within any of the areas of interest, was manually removed from each trial. We used EyeLink Data Viewer (SR Research Ltd.) to analyze the results and perform a standard fixation cleaning procedure. Fixations lasting 80 ms or less were merged with neighboring fixations lasting more than 80 ms and within a distance of 0.5 visual degrees from the original fixation. A similar procedure was performed for fixations lasting of 40 ms duration or less, with a merging distance of 1.25 visual degrees. A total of 448 fixations were merged as a result of these steps. Additionally, all fixations outside the zones of interest were removed, resulting in the exclusion of 10,382 fixations.

Data analysis was performed with linear mixed-effects regression models in R 4.3.1 (43) using the lme4-package (version 1.1-34) (6). The p-values for fixed effects were obtained using the lmerTest-package (version 3.1-3) with Satterthwaite’s degrees of freedom approximation (29) for linear mixed-effects regression models.

Linear mixed-effects models were constructed for each variable. The fixed effect of pun presence or absence in the poster (pun vs no pun) was included in all models. Additionally, in the model for total dwell time on posters and the number of switches between text and image, the fixed effect of human image presence or absence on the non-verbal part of the poster (human vs no human) was included to evaluate if this factor had any extra impact on poster viewing. Participants were included in the model as a random effect. Visual inspection of residual plots did not reveal any obvious deviations from homoscedasticity or normality.

During preliminary data processing, we visually assessed the normality of distributions of all variables using distribution plots. Unfortunately, we found that all variables had non-normal distributions. Outliers were identified using the interquartile range (IQR) method: outliers = observations > third quartile + 1.5 * IQR < first quartile - 1.5 * IQR. Outliers, making up no more than 5% of all observations for each variable, were excluded. However, for the “number of regressions to headline” variable, outliers were not excluded as they constituted 40% of all observations. Table 2 presents the mean values for all variables.

**Table 2.** Mean values (M) across variables for posters with and without a pun, as well as the standard deviations (SD).

	Posters with a pun		Posters without a pun	
	M	SD	M	SD
Total dwell time (ms)	5674	2107	5939	2006
Gaze switches count between text and picture (N)	4.7	2.2	4.7	2.3
Total dwell time on the picture (ms)	1903	1169	1803	1097
Fixation count on the picture (N)	7.4	4.4	7.7	4.7
Total dwell time on the headline (ms)	1423	807	1415	707
First run dwell time on the headline (ms)	624	294	639	350
Regression count to the headline (N)	0.95	0.93	0.98	0.97
Total dwell time on the main text area (ms)	1664	775	1573	792
Total dwell time on the brand name (ms)	689	373	659	349

The statistical analysis showed a significant effect of the presence of a pun in the poster (F(1, 1189) = 2.412, p = 0.0160) on total dwell time on posters, with longer dwell time associated with the absence of a pun. No significant effect of the presence of a human image on poster dwell time was found ((F(1, 1189) = 1.841; p = 0.0658). We found no significant effect of both analyzed factors on the number of gaze switches between the picture area and the text area (for pun presence F(1, 1185) = 0.0816; p = 0.7752; for image of a human presence F(1, 1184) = 2.8982; p = 0.0889). No significant effect of pun presence was observed for dwell time on the picture (F(1, 1158) = 2.5836; p = 0.1082), as well as for the number of fixations on the picture (F(1, 1177) = 1.701; p = 0.1924). No effect of pun presence is observed on dwell time on headlines (F(1, 1174 ) = 0.0458; p = 0.8306) and brand names (F(1, 1173) = 2.0463; p = 0.1528). A significant effect of the presence of a pun in the poster is found for the dwell time on the main text area (F(1, 1182) = 4.5659; p = 0.03282) with longer dwell time for posters with a pun. The analysis did not show any effect of the presence of a pun on the first run dwell time on headlines (F(1, 1146) = 1.5748; p = 0.2098), as well as on the regression count to the headline (F(1, 1218) = 0.4069; p = 0.5237).

Readers read text on the advertising posters twice as long as they looked at the picture (mean dwell time on text areas in total is 3703 ms vs. mean dwell time on the picture is 1847 ms). This result is in line with numerous studies (40; 45). We can build a general hierarchy of the distribution of human attention on the studied advertising posters based on the mean time that participants spent viewing the areas of interest:

picture (1847 ms) -> main text area (1613 ms) -> headline area (1418 ms) -> brand name area (672 ms)

Our analysis showed that in 86% of cases (1094 out of 1270 trials), the first fixation on the poster fell on the text. In 88% out of those (972 trials), it fell on the headline. Then the participants read the main advertising text (the second fixation fell on the second advertising text in 58% of cases, 738 trials) and then briefly examined the picture.

## Discussion

Our study examined how people process posters containing puns, created using a combination of images and textual elements of the poster, compared to posters without puns. In posters with puns, the headline contains a collocation, and the main textual area and the image add an alternative collocation meaning or support the idiomatic collocation meaning. The results of the preliminary experiment (27) showed that posters with and without puns were rated equally on the comprehensibility scale, and posters with puns are rated as significantly more attractive, original, effective, and positive compared to posters without puns. Thus, it can be assumed that ambiguity in the posters was correctly resolved by the participants of the experiment, and the creators of the advertisements correctly predicted how these puns would be perceived.

The results of eye-tracking experiment showed that participants viewed posters without puns significantly longer than posters with puns. This result is unexpected and may be related to the design of the experiment, namely that participants viewed the posters with and without puns in a random order, and perhaps tried to find puns where there were none.

We did not find any significant difference in reading headlines with and without a pun, both at the early stages of processing and on a global scale. Previous studies (see 8 for a review) have shown that idioms are read faster than the control phrases. Since our poster headlines with and without puns did not differ in length, one would expect that headlines with collocations would be processed faster, but this was not the case. This lack of differences may be because readers either did not notice that the collocation can be understood in different ways or did not find it difficult to understand. In favor of the second explanation is also the fact that readers did not make more regressions to the headline with a pun. We also found no significant differences in brand name processing for posters with and without puns. For both types of posters, the brand name area attracted the least attention of all AOIs.

Participants spent significantly more time reading the main text area of the posters with puns than the main text of the posters without puns. Because the alternative meaning of the collocation (often literal) is added in the main text, the increased reading time in this area may indicate that participants understood the pun in the headline in one sense, and when confronted with the alternative meaning of the pun, they had to make some effort to connect the two meanings, as evidenced by the increased reading time in the main text area. This indicator may reflect the process of pun resolution, because pun resolution requires cognitive effort (52), thus increasing reading time. Total dwell time on an AOI is reported to be an indicator of the organizing process, when readers make connections between words or images in order to create a coherent verbal or pictorial mental representation (3).

We were most interested in how readers integrate information from text and images, which can be quantified as the number of switches between areas of interest (3). These switches may be interpreted as attempts to combine and integrate text and images (21). We expected that the presence of a pun supported by a picture and text would encourage participants to switch between these areas more frequently to understand the pun itself, but this pattern was not observed. Our results showed that readers were no more likely to switch between text and picture on posters with puns than on posters without puns. This can be explained by two reasons: a) the puns in our material were simple enough that participants did not need to go back to already viewed parts of the poster to find semantic connections; b) participants tried to find puns where there were none.

When analyzing the total dwell time on advertising posters and the number of switches between the text and the picture of the poster, we took into account the factor of the presence or absence of a human image on the poster, since, as described above, the presence of a human image in the visual stimulus significantly affects its viewing. We expected that when viewing a visual stimulus in which an image of a human is present, the viewer will look at the human for a longer time. Additionally, it was expected that viewers would allocate more attention to posters featuring human images compared to those featuring inanimate objects. We found no significant effect of the presence of a human image on any of eye-tracking measures. The participants did not pay more attention to the portraits. It contradicts the data received by (56; 40). This issue requires further consideration. We assume that our findings may be attributed to the features of the advertising posters employed in this study, specifically their non-verbal component primarily being conveyed by one object, such as a person, animal or object. If the non-verbal component included a range of objects, the image of a human would likely receive the most attention.

We also revealed that visual information (pictures) in the advertising posters was processed faster and with smaller number of fixations than text components. An image is believed to play a dominant role in an advertising poster (25), draw attention to the advertisement (41), and is better remembered (15; 45). Moreover, because readers do not need to make multiple fixations to make sense of the pictures (36; 45), participants expend fewer cognitive resources to view them, i.e., they view/examine them faster than they read the advertising text. These findings are supported by the results of our study.

In our study, we found out that participants spent twice as much time reading the text of the posters as they did viewing the image, regardless of whether the poster was with or without a pun. This finding supports the results of (45), but differs from the evidence provided by (44; Rayner et al., 2008) with similar experimental instructions. In these studies, the participants were also asked to evaluate the advertising posters according to several parameters, and they spent significantly longer looking at the picture than reading the text. A possible explanation is that people tend to engage in reading the text because our prior experience tells us that text often conveys significant and meaningful information, therefore, it is considered potentially important (17).

Furthermore, the participants' initial interaction with the posters began with reading the text. That is, participants began viewing the posters by reading the most important/key text (i.e., headline), moved to smaller text, and only then moved to the picture. Thus, the participants started viewing the posters from the top, moved downward, and their attention moved in a bottom-up fashion from larger text to smaller text. A similar pattern is described in (45). This finding is at odds with the observation by (37; 54) who showed that viewing a polycode text begins with the processing of a nonverbal element.

Our results suggest differences in processing of advertisements at the low levels (eye-movements) and high levels (evaluation). Readers/viewers do not process posters with puns longer than posters without puns, although they do rate them as more attractive, original, effective, and positive (27). Thus, readers/viewers notice the presence of puns in posters, which leads to higher subjective evaluations of these posters, but does not affect the way readers examine these posters. The only eye-tracking measure confirming that readers notice the pun is an increase in reading time for the main text area, where the alternative collocation meaning is added. The obtained results indicate that there are no strong correlations between the participants' subjective evaluation of posters and the eye movement indicators, and the results are consistent with the findings of other researchers (4).

## Conclusion

This study examines advertising posters in which a pun is created using all the components of a polycode text, both verbal and pictorial.

Our findings pertaining to the first research question (What is the impact of puns in advertising posters on eye movement patterns?) do not align with prior research. We revealed that the presence of a pun, crafted using a combination of pictorial and textual elements of an advertising poster does not change the overall viewing pattern of the poster, does not draw more viewers` attention, and does not induce viewers to switch their gaze from the text to the image.

Regarding the second question of whether there is a difference in how verbal and non-verbal parts of advertisements are viewed, our findings from the eye-tracking experiment indicated that the reading of advertisements was primarily text-directed. We found that readers begin processing polycode advertisements with text and spend more time reading text than viewing images. The presence of a pun in advertising, where both verbal and non-verbal elements contribute to the intended meaning, does not prompt viewers to switch their gaze between text and image.

In terms of the third research question, our findings did not demonstrate any significant differences in the low-level processing of posters with puns compared to those without. Though there are differences at the level of comprehension and subjective evaluation of advertising posters. Posters with puns are rated higher, but these higher evaluations are not the result of any particular viewing pattern for posters with puns.

Based on our research, we can make some recommendations for advertising poster designers:

1. It is advisable to incorporate puns into the advertising posters as they serve multiple purposes. Firstly, the ambiguity in the slogan of an advertising poster encourages a more thorough examination of main text area, which refers to the area where the primary advertising text is located. Secondly, puns enhance the recognition of the poster, leading to a more favorable subjective assessment and increasing the likelihood of attracting visual attention.

2. When designing an advertising poster that contains only one image element, it is unnecessary to select a portrait or image of a person as this element. Research indicates that there is no significant difference in viewing posters featuring faces and those featuring other visual elements.

3. When constructing advertising posters, it is advisable to allocate extra consideration to the textual component, particularly when formulating the title. This section receives initial attention and subsequent revisits, contrary to the conventional belief that the visual processing of a multimodal text commences with an image.

It is also important to acknowledge the limitations of the present study. There was a gender imbalance among the participants (41 female out of 53 participants whose eye movement were recorded). Further research is necessary to verify the observational results and extend them to the population level. Furthermore, it is important to consider the individual characteristics of participants that could affect the experiment's outcomes, including their interests, past experiences, attitudes towards advertising, skills and knowledge, memory capacity, and others. Another limitation is that the present paper illustrates viewing behavior throughout laboratory tasks that may be significantly different from ad viewing in the real world. Besides, only a limited number of advertising posters were examined in this paper, and all of them have the same structure: three text zones on the right half of a poster and a picture on the left half. This experiment does not allow us to explore the full breadth of the observed phenomenon, namely, the richness and variety of alternative forms of the advertisements. Thus, it remains uncertain how the use of different samples of advertisements may affect the results. Future research could include a larger sample of polycode advertisements to investigate and generalize our findings related to the viewing processes of pun-filled posters. In addition, the AOI sizes for the verbal and pictorial parts of the posters varied slightly across different stimuli in the experiment, which may have affected the calculation of some eye movement measures.

Despite these limitations, our findings help to identify how attention is distributed between verbal and non-verbal components of polycode texts, as well as to explore the effect of a pun in a headline on the navigation decisions, and identify the type of a poster that is easy and convenient for retrieving information at both low and high levels of processing. The data obtained offers opportunities for further investigation of the reading process, means of resolving ambiguity in various types of texts and the relations between verbal and non-verbal parts of a polycode poster.

### Ethics and Conflict of Interest

The authors declare that the contents of the article are in agreement with the ethics described in http://biblio.unibe.ch/portale/elibrary/BOP/jemr/ethics.html and that there is no conflict of interest regarding the publication of this paper.

### Acknowledgements

This research was supported by Russian Science Foundation grant No 21-18-00429 “Cognitive mechanisms of multimodal information processing: text type & type of recipient”.

We wish to thank all the participants who took part in the experiment. We are grateful to Alena Konina for her helpful comments and editing the English text and to Ekaterina Saenko for the help with the data analysis.
